# Sparse Modeling Reveals miRNA Signatures for Diagnostics of Inflammatory Bowel Disease

**DOI:** 10.1371/journal.pone.0140155

**Published:** 2015-10-14

**Authors:** Matthias Hübenthal, Georg Hemmrich-Stanisak, Frauke Degenhardt, Silke Szymczak, Zhipei Du, Abdou Elsharawy, Andreas Keller, Stefan Schreiber, Andre Franke

**Affiliations:** 1 Institute of Clinical Molecular Biology, Christian-Albrechts-University of Kiel, Kiel, Germany; 2 Chemistry Department, Division of Biochemistry, Faculty of Sciences, Damietta University, New Damietta City, Egypt; 3 Chair for Clinical Bioinformatics, Saarland University, Saarbruücken, Germany; 4 Department of Internal Medicine I, University Hospital Schleswig-Holstein, Kiel, Germany; INSERM, FRANCE

## Abstract

The diagnosis of inflammatory bowel disease (IBD) still remains a clinical challenge and the most accurate diagnostic procedure is a combination of clinical tests including invasive endoscopy. In this study we evaluated whether systematic miRNA expression profiling, in conjunction with machine learning techniques, is suitable as a non-invasive test for the major IBD phenotypes (Crohn's disease (CD) and ulcerative colitis (UC)). Based on microarray technology, expression levels of 863 miRNAs were determined for whole blood samples from 40 CD and 36 UC patients and compared to data from 38 healthy controls (HC). To further discriminate between disease-specific and general inflammation we included miRNA expression data from other inflammatory diseases (inflammation controls (IC): 24 chronic obstructive pulmonary disease (COPD), 23 multiple sclerosis, 38 pancreatitis and 45 sarcoidosis cases) as well as 70 healthy controls from previous studies. Classification problems considering 2, 3 or 4 groups were solved using different types of penalized support vector machines (SVMs). The resulting models were assessed regarding sparsity and performance and a subset was selected for further investigation. Measured by the area under the ROC curve (AUC) the corresponding median holdout-validated accuracy was estimated as ranging from 0.75 to 1.00 (including IC) and 0.89 to 0.98 (excluding IC), respectively. In combination, the corresponding models provide tools for the distinction of CD and UC as well as CD, UC and HC with expected classification error rates of 3.1 and 3.3%, respectively. These results were obtained by incorporating not more than 16 distinct miRNAs. Validated target genes of these miRNAs have been previously described as being related to IBD. For others we observed significant enrichment for IBD susceptibility loci identified in earlier GWAS. These results suggest that the proposed miRNA signature is of relevance for the etiology of IBD. Its diagnostic value, however, should be further evaluated in large, independent, clinically well characterized cohorts.

## Introduction

Inflammatory bowel disease (IBD) is a complex, polygenic, chronic intestinal disorder of unknown etiology, comprising two major types: Crohn’s disease (CD) and ulcerative colitis (UC). IBD is believed to evolve through a dysregulated response of the immune system to the commensal microbiota associated with intestinal tissues in a genetically susceptible host. The diagnosis of IBD is often achieved only months or years after the first onset of symptoms and still requires a multitude of information from clinical, radiological, endoscopic and histological tests. Extensive heterogeneity is observed in terms of disease presentation, behavior, and response to treatment. However, a definite diagnosis of CD or UC cannot be established in approximately 10%–17% of colitis patients (known as “indeterminate colitis” (IC)) [1] and more than 10% of IBD patients change diagnosis (CD or UC) during the first year of the disease course [2]. Fecal and serological diagnostic tests, e.g. for calprotectin, lactoferrin or CRP (C-reactive protein) as well as serum antibodies like pANCAs (perinuclear antineutrophil cytoplasmic antibody) and ASCAs (anti-S.cerevisiae antibody), supplement invasive endoscopic/colonoscopic methods to verify IBD-diagnosis, to differentiate between the major subtypes or to evaluate disease progression [3,4]. In the last 10 years, several genome-wide association studies (GWAS) were carried out to identify common susceptibility variants for IBD. In a large meta-analysis of previous IBD GWAS, including more than 75,000 cases and controls, Jostins *et al*. identified 71 additional loci, increasing the total number of known IBD susceptibility loci with association of genome-wide significance to more than 163 [5]. While GWAS findings have added tremendously to the understanding of disease etiology and the genetic architecture, common genetic variants have low diagnostic value as shown for IBD [6] and other diseases [7]. Other studies, employing mRNA-based measurements of differential gene expression in tissue or peripheral blood of IBD patients of varying disease state, revealed distinct expression patterns [8–11]. Limitations of these studies were reported when comparing cases and healthy controls or trying to classify disease subphenotypes [12]. Non-coding, regulatory microRNAs (miRNAs) have been studied in the context of their function in IBD [13] but especially because of their ability to serve as diagnostic markers, as recently summarized by Chen *et al*. [14]. As miRNA expression levels are more stable in tissues and body fluids, such as peripheral blood, and as miRNAs act as master-regulators of mRNAs, differential signatures of miRNAs could serve as superior, non-invasive diagnostic markers to verify IBD diagnosis, discriminate between major IBD subphenotypes and to predict prognosis. A core set of deregulated miRNAs has been identified in a series of studies investigating differential miRNA expression in biopsies and peripheral blood of IBD patients [15–22]. Functional links gained from the analysis of IBD-associated miRNA target genes implicate an involvement of cellular pathways of the immune system (NF-κB, IL-23/IL-23R, IL-6/STAT3) [23–29], autophagy [13,30,31], epithelial barrier function [32,33], IBD-associated dysplasia and colorectal cancer [34–36] in IBD disease etiology. Besides these mechanistic insights into the disease, highly accurate predictive sets of miRNAs suitable for diagnostic purposes have not yet been reported. Interestingly, most of the afore-described studies, investigating deregulation of miRNAs, follow the classic approach of statistical hypothesis testing for significant differential expression of single candidate miRNAs. Some publications, however, point out an alternative way of employing large miRNA datasets and machine-learning techniques, such as support vector machines (SVMs) [37] or random forests (RFs) [38]. Keller and colleagues successfully applied SVM-based approaches to identify diagnostic miRNA-profiles for several different diseases [39], such as multiple sclerosis [40,41], lung cancer [42] or male infertility [43]. Others used similar analysis strategies to generate miRNA expression signatures for pharyngeal squamous cell carcinomas [44], thyroid lesions [45], lung adenocarcinoma [46] or pulmonary tuberculosis [47]. Even ulcerative colitis has been investigated using SVMs, leading to a signature of platelet-derived miRNAs [48].

Here we investigate microarray-based miRNA expression profiles from peripheral blood of IBD patients, using penalized SVMs [49] and random forests for distinction of CD and UC from healthy controls and other complex inflammatory diseases (chronic obstructive pulmonary disease (COPD), multiple sclerosis, pancreatitis and sarcoidosis). The promising results of our pilot study show, that machine-learning techniques and miRNA signatures should be further investigated for IBD diagnostics. Moreover, the miRNA profiles identified yield further insight into the disease-relevant signaling pathways.

## Material and Methods

### Patient recruitment and sampling

Clinical data and sample material used in this study were obtained under written informed consent of patients as well as healthy donors, and under approvals of the local ethics committees (Biobank Popgen & Ethik-Komission der Medizinischen Fakultät, Universitätsklinikum Schleswig-Holstein, Kiel). We randomly selected blood samples of 40 CD, 36 UC patients and included 38 healthy controls (HC) from our biobank. Patients were collected at the UKSH tertiary referral center. Diagnoses were verified by a clinician after reviewing the respective medical health records. As shown in **Table 1**, patients of every group were matched regarding demographic parameters (mean age at diagnosis of 27.3 and 28.1 years for CD and UC cases, respectively; mean age at sampling of 46.0 and 43.8 years for CD and UC cases, respectively; fraction of males of 54.1% in CD and 53.1% in UC patients, respectively). The majority of the patients was treated with anti-TNF-α inhibitors, such as Infliximab or Mesalazine (67.6% of CD and 90.6% of UC cases) and is therefore assumed to be stable regarding the clinical presentation. The activity of immune cells is assumed to be altered partially since a fraction of the patients additionally was treated with immunosuppressive drugs, such as Azathioprine, Cyclosporine, 6-Mercaptopurine or Tacrolimus (48.6% of CD and 31.3% of UC cases). Furthermore a substantial fraction of the patients underwent the clinically common treatment with SAIDs (steroidal anti-inflammatory drugs; 29.7% of CD and 56.3% of the UC cases, respectively) and/or NSAIDs (non-steroidal anti-inflammatory drugs; 2.7% of CD and 6.3% of the UC cases, respectively). However, based on the available data exacerbation of IBD at sampling was ruled out for 51.4% of CD and 56.3% of UC cases.

**Table 1:** Characterization of the study subjects. Grouped by CD, UC and HC frequency information (in percent) on demographics (gender and smoking status), medication (anti-TNF-α, immunosuppressant, SAIDs and NSAIDs) as well as symptoms (disease attack at sampling, stenosis, fistula and surgery) is shown.

		demographics		medication				symptoms			
		male	smoker	anti-TNF-alpha	immunosuppressant	said	nsaid	disease attack at sampling	stenosis	fistula	surgery
**CD**	**no**	45.9	35.1	32.4	51.4	70.3	97.3	51.4	27.0	48.6	29.7
** **	**yes**	54.1	64.9	67.6	48.6	29.7	2.7	0.0	62.2	48.6	70.3
** **	**NA**	0.0	0.0	0.0	0.0	0.0	0.0	48.6	10.8	2.7	0.0
**UC**	**no**	46.9	68.8	9.4	68.8	43.8	93.8	56.3	78.1	84.4	90.6
** **	**yes**	53.1	31.3	90.6	31.3	56.3	6.3	0.0	3.1	3.1	3.1
** **	**NA**	0.0	0.0	0.0	0.0	0.0	0.0	43.8	18.8	12.5	6.3
**HC**	**no**	46.9	0.0	0.0	0.0	0.0	0.0	0.0	0.0	0.0	0.0
** **	**yes**	53.1	0.0	0.0	0.0	0.0	0.0	0.0	0.0	0.0	0.0
** **	**NA**	0.0	100.0	100.0	100.0	100.0	100.0	100.0	100.0	100.0	100.0

### miRNA extraction and microarray measurement

After sampling, peripheral blood was anticoagulated using ethylenediaminetetraacetic acid (EDTA) and immediately processed for RNA isolation. Total RNA, including miRNAs, was extracted using the miRNeasy Mini Kit (Qiagen GmbH, Hilden, Germany) and stored at -80°C. All samples were analyzed on the automated Geniom Real Time Analyzer (GRTA, febit biomed GmbH, Heidelberg, Germany) using the Geniom miRNA Biochip for Homo sapiens, covering 866 human miRNA species [50]. Since miRBase has been updated from version 12 to 14 during the time course of the study, we used 863 miRNAs that were consistently present in all three versions for the final data analysis. Biotin labeling was conducted by microfluidic enzymatic on-chip labeling of miRNAs as described previously [51]. Hybridization was carried out for 16 hours at 42°C followed by signal enhancement processing with GRTA. Detection images were analyzed using the Geniom Wizard Software.

### Data preprocessing

Sample data for other inflammatory diseases, representing the inflammation control panel for the current investigation, was taken from a previously published study [39]. This dataset comprised 24 COPD, 23 multiple sclerosis, 38 pancreatitis and 45 sarcoidosis cases as well as another 70 healthy controls. Raw data of these samples was downloaded from Gene Expression Omnibus (GEO, Accession code: GSE31568) and analyzed jointly with raw data of samples generated for this study. Samples with median background-subtracted intensity exceeding 1.5⋅*IQR* where removed as outliers resulting in 273 samples, including 37 CD, 32 UC, 92 HC, 23 COPD, 23 multiple sclerosis, 35 pancreatitis and 32 sarcoidosis cases. To account for batch effects arising from differences in the source of data the background-subtracted intensity values were centered with regard to the medians of the healthy controls. Normalization then was performed using the R package vsn [52] for robust calibration and variance stabilization.

### Classification with penalized support vector machines

To obtain mathematical models that allow diagnostic applications as well as the elucidation of the role of miRNAs in the development of IBD, different types of classification problems were investigated. Aiming for the distinction between CD, UC and HC initially a set of models considering 2 groups was examined (CD vs. HC, UC vs. HC, CD vs. UC). Classification problems additionally incorporating IC (CD vs. IC, UC vs. IC, IC vs. HC) were carried out to differentiate CD, UC and HC from general inflammation. Models aiming for the distinction of combinations of groups were examined by jointly considering 3 groups (CD vs. UC+HC, UC vs. CD+HC, HC vs. CD+UC as well as CD vs. UC+IC, UC vs. CD+IC, IC vs. CD+UC). Finally, also a set of models allowing for 4 groups was investigated (CD vs. UC+HC+IC, UC vs. CD+HC+IC, HC vs. CD+UC+IC, IC vs. CD+UC+HC). Each of the 16 classification problems was solved using different types of linear penalized support vector machines, namely LASSO SVM, elastic net SVM, SCAD SVM and elastic SCAD SVM. Additionally, the linear standard SVM not performing feature selection was used as a reference. It is worth noting that not every classification problem considered has a diagnostic meaning. However, for the subsequent construction of combined classifiers, none of these can be neglected.

Support vector machines (SVMs) are widely used for solving supervised classification problems. However, SVMs do not allow for the selection of important variables (feature selection). Applying the mathematical idea of regularization abolishes this limitation [49]. Accordingly, regularized incarnations of the standard SVM along with efficient algorithms for optimizing their objective functions have been proposed. All these methods share the use of penalties for model complexity to provide sparse solutions, i.e. small sets of features that enable good classification.

For data $\text{D}\in {\left\{({\text{x}}_{\text{i}},{\text{y}}_{\text{i}})|{\text{x}}_{\text{i}}\in {\text{R}}^{\text{m}},{\text{y}}_{\text{i}}\in \{-1,\;1\}\right\}}_{\text{i}=1}^{\text{n}}$ with input vectors x_i_ and class labels y_i_ for i = 1, …, n the SVM optimization problem corresponds to the minimization of ${\left\|\bar{\beta }\right\|}_{2}^{2}$ with respect to the decision rule ${\text{y}}_{\text{i}}\left(\bar{\beta }\cdot {\text{x}}_{\text{i}}-\beta_{0}\right)\geq 1$. As shown by Hastie [49] this also can be written as a regularization problem ${\text{min}}_{\beta_{0},\bar{\beta }}\;\sum_{\text{i}=1}^{\text{n}}\;\text{L}({\text{y}}_{\text{i}};\text{f}({\text{x}}_{\text{i}}))+{\text{p}}_{\lambda }\left(\bar{\beta }\right)$ where $\text{L}\left({\text{y}}_{\text{i}};\text{f}({\text{x}}_{\text{i}})\right)=\text{max}\left(0,1-{\text{y}}_{\text{i}}(\bar{\beta }\cdot {\text{x}}_{\text{i}}-\beta_{0})\right)$ is a loss (or cost) function and ${\text{p}}_{\lambda }\left(\bar{\beta }\right)$ a penalty function with parameter λ. The classic choices of ${\text{p}}_{\lambda }\left(\bar{\beta }\right)$ include the ridge penalty [49] (standard SVM, ${\text{p}}_{\lambda }\left(\bar{\beta }\right)=\lambda {\left\|\bar{\beta }\right\|}_{2}^{2}$) and the LASSO [53] (least absolute shrinkage and selection operator, ${\text{p}}_{\lambda }\left(\bar{\beta }\right)=\lambda {\left\|\bar{\beta }\right\|}_{1}$) as well as their combination known as the elastic net [54] (${\text{p}}_{\lambda }\left(\bar{\beta }\right)=\lambda_{1}{\left\|\bar{\beta }\right\|}_{1}+\lambda_{2}{\left\|\bar{\beta }\right\|}_{2}^{2},\;\lambda_{1},\lambda_{1}\geq 0$). More recently a penalty function improving the properties of the LASSO was published. The SCAD (smoothly clipped absolute deviation) penalty [55] is given by the quadratic spline

$${\text{p}}_{\lambda }^{\text{SCAD}}\left(\bar{\beta }\right)=\sum_{\text{j}=1}^{\text{m}}\left(\lambda \left|\beta_{\text{j}}\right|\text{I}\left(\left|\beta_{\text{j}}\right|\leq \lambda \right)+\frac{{\left|\beta_{\text{j}}\right|}^{2}-2\text{a}\lambda \left|\beta_{\text{j}}\right|+\lambda^{2}}{2\left(\text{a}-1\right)}\text{I}\left(\lambda <\left|\beta_{\text{j}}\right|\leq \text{a}\lambda \right)+\frac{\left(\text{a}+1\right)\lambda^{2}}{2}\text{I}\left(\left|\beta_{\text{j}}\right|>\text{a}\lambda \right)\right)$$

for a > 2 and λ > 0 and indicator function I(⋅). Similar to the LASSO this function provides feature selection by shrinking small coefficients |β_j_| ≤ λ to zero (resulting in a sparse model). However, in contrast to the LASSO it applies a constant penalty to large coefficients |β_j_| > aλ (resulting in an approximately unbiased model). Combining SCAD with the ridge penalty finally results in the elastic SCAD penalty [56] defined as $\text{p}\left(\bar{\beta }\right)={\text{p}}_{\lambda_{1}}^{\text{SCAD}}\left(\bar{\beta }\right)+\lambda_{2}{\left\|\bar{\beta }\right\|}_{2}^{2}$ with tuning parameters λ_1_, λ_2_ ≥ 0. Efficient implementations of SVMs regularized using the penalty functions mentioned before are available in the R package penalizedSVM (version 1.1) [57].

The normalized miRNA expression data were randomly split at a ratio of 5:3, preserving the proportion of samples per group. The first partition was used to construct the respective model, whereas the second was used for evaluation. To estimate the distribution of each model’s predictive performance, the partitioning was conducted repeatedly applying 500-fold holdout sampling (random choice of samples without replacement). The tuning parameters thereby were trained using 5-fold cross validation and fixed grid search based on the respective training datasets. The final SVMs then were obtained by selecting the sparsest median performing model for each investigated classification task. A classifier’s performance thereby was measured by the area under the receiver operating characteristic (ROC) curve (AUC, sensitivity as a function of 1-specificity). For illustrative purpose, additional performance measures of varying informational content were determined, e.g. balanced accuracy (BAC), sensitivity (SN), specificity (SP). For each classification problem the sets of miRNAs (miRNA signatures) considered by the sparsest median performing model were selected for further investigation, including validation with random forests and target enrichment analysis.

According to the principal of majority voting [58], the selected models were used to construct combined classifiers for exemplary diagnostic problems (CD vs. UC, CD vs. UC vs. HC, CD vs. UC vs. IC and CD vs. UC vs. HC vs. IC). The diagnoses provided by these models were evaluated using the classification error rate estimated based on the complete dataset. Finally, the risk of observing small combined error rates by chance was assessed using the Z-statistic with parameters estimated based on 1,000-fold permutation of the class labels. Corresponding p-values were calculated using the normal cumulative distribution function and tested for significance using the standard significance level of 0.05.

### Validation with random forests

A second machine-learning approach, random forest (RF), was used to analyze the reported miRNA dataset. RF is an ensemble tree method that was first introduced by Breiman *et al*. in 2001 [38], and has been shown to be accurate in both classification and regression problems. Randomization is introduced by constructing each decision tree with a randomly chosen bootstrap sample. Additionally, at each node the optimal splitting variable is selected among a random subset of variables (predictors). Variables selected in RF classification trees are assigned an importance score that is a measure of how much the particular predictor contributes to classifying the respective data. In this study relative recurrency variable importance metric (r2VIM), recently proposed as a measure of variable importance, was used. Based on the permutation importance scheme this measure reduces noisy signal selection [59]. For further details on the concept of RF refer to Strobl *et al*. [60]).

To evaluate the validity of the feature selection employed by the penalized SVMs, two random forests were built for each classification problem. While the first model incorporated variables per holdout selected by the RF, the second contained variables per holdout selected by the SVM (holdout signature). To further validate the meaningfulness of the proposed miRNA signature, another two random forests were built. This time the variable set was constant across the training datasets for each classification problem. For the third model all variables selected in at least one training dataset were ranked by the number of times they were selected and the 50% most frequently selected variables (top signature) were used for training of the RF. Finally, for the fourth model the variables incorporated by the sparsest median performing SVM (median signature) were used.

For comparability, model training, as well as evaluation, incorporated the randomly selected datasets (500-fold holdout partitioning) previously used to construct the SVM classifier. As a measure of model performance again the area under the ROC curve (AUC) was used. All RF analyses were performed in R (version 3.0.1) using the packages parallelRandomForest (version 4.6–7) and ROCR (version 1.0–5) [61]. For each forest, 500 trees (ntree) were built with a terminal node size (nodesize) of 10% of the sample size. The number of randomly selected variables at each node (mtry) was set to the square root of the total number of predictors. For each analysis a random seed was set to a randomly chosen number between 1 and 100,000.

### miRNA target gene enrichment analysis

Experimentally validated miRNA target genes were extracted from miRTarBase [62] version 4.5 and tested for significant enrichment within the previously published IBD susceptibility loci [5]. In total more than 163 genetic risk loci have been previously identified as being associated with inflammatory bowel disease (CD: 30, UC: 23, IBD: 110) [5]. 49 out of 1332 experimentally validated miRNA target genes, as listed in miRTarBase, overlap with these loci. To test for overrepresentation of risk loci among the targets of the miRNAs selected for distinguishing CD, UC and HC, Fisher’s exact test was applied (see also **S7 Table**). Enrichment was considered as being significant in case p-values were smaller than 0.05. Adjustment for multiple testing was conducted using Bonferroni correction.

## Results

### Differential expression analysis of peripheral blood miRNAs

To examine potential deregulation, we analyzed expression levels of 863 miRNAs in 40 Crohn’s disease patients, 36 ulcerative colitis patients as well as 70 healthy control individuals. After RNA isolation from freshly drawn peripheral blood, miRNA expression data were generated utilizing the Geniom Biochip miRNA (Homo sapiens). After batch-correction and normalization the background-subtracted microarray intensity values did not show considerable sample-based mean-variance dependencies or sample-based variability of dispersion estimates. As illustrated using multidimensional scaling based on Spearman’s rank correlation distance (**S1 Fig**), the groups of interest are visually hardly distinguishable.

In the differential expression analysis (summarized in **S1 Table)** we were able to identify 292 and 353 miRNAs as being significantly deregulated in CD and UC, respectively, when compared to healthy controls (Student's t-test with significance threshold of 0.05 applied to p-values adjusted for multiple testing according to Holm’s sequential Bonferroni method). In terms of miRNA expression level differences these results correspond well to previously published findings (see **S2 Fig** and **S2 Table**). The degree of consistency, thereby, increases with the sample size of the reference study. The correspondence to expression levels of the core set of altered miRNAs involved in IBD [14] was estimated to be 75.0%. Additional 20 miRNAs investigated by Wu *et al*. (14 cases of active CD, 10 cases of active UC, 13 HC) agree with our data in 45.0% of the cases [16]. Evaluation of another 7 miRNAs identified in a study employing 20 UC and 20 HC samples shows a correspondence of 71.4% [48]. Finally, the studies conducted by Zahm *et al*. [18] (11 deregulated miRNAs identified in 46 cases of active CD and 32 HC) and Paraskevi *et al*. [22] (17 miRNAs, 128 cases of active CD, 88 cases of active UC, 162 HC) completely overlap with our results (correspondence of 100.0%). Interestingly, a large proportion of miRNAs that have previously been reported as being differentially expressed only for a certain group (CD or UC) appear to be deregulated similarly in both subtypes in our data and thus may be general IBD miRNAs. This effect may be explained due to the smaller sample size and/or higher variability in previous studies.

### Classification with penalized support vector machines

Since there are various ways to construct complex classifiers for the distinction between CD, UC and HC (and IC, respectively), we assessed different types of penalized SVMs as well as the corresponding sets of miRNAs based on model performance and sparsity. Considering models incorporating 2 groups, differences in holdout-based median classifier performance of the penalization methods were small. However, due to its theoretic properties, the elastic SCAD SVM (median AUC = 0.97) was chosen for further investigation. Plots and tables illustrating the performance of the LASSO SVM (median AUC = 0.96), elastic net SVM (median AUC = 0.94) and SCAD SVM (median AUC = 0.95) are shown in **S4–S6 Figs** and S3–S5 **Tables.**

**Fig 1:**
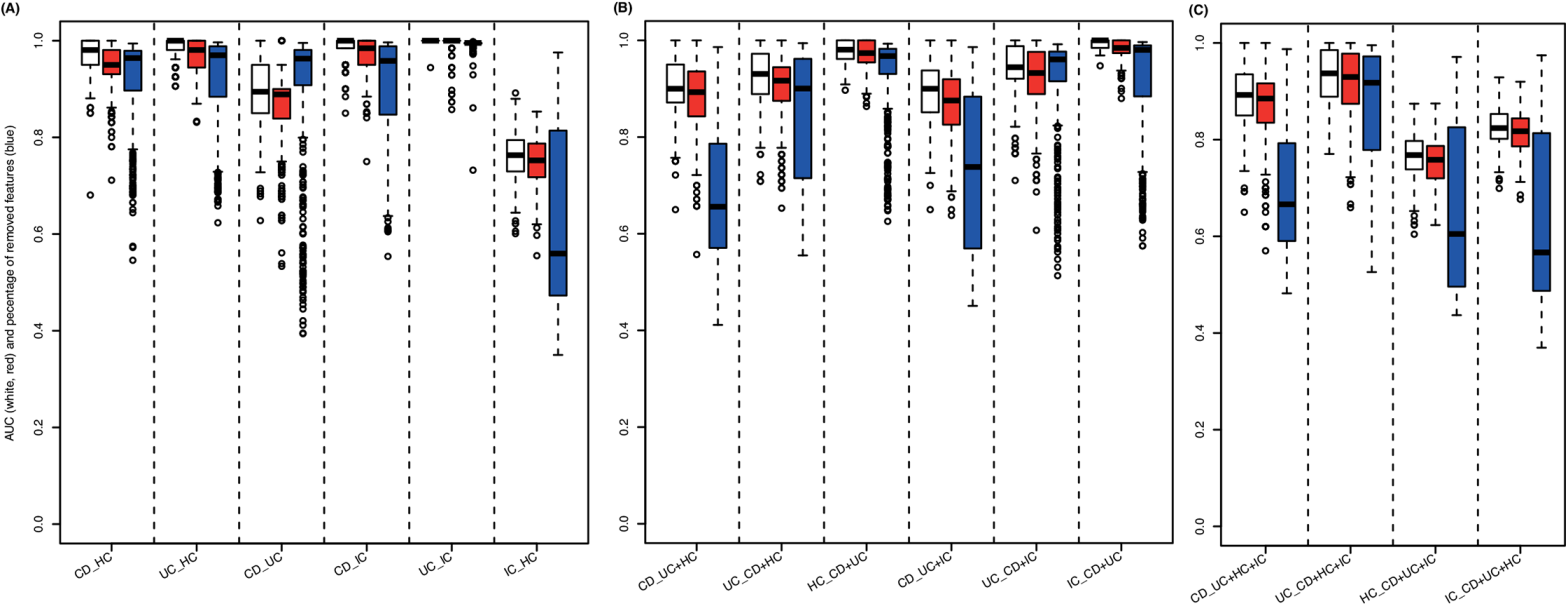
SVM classification results. Measured by the area under the ROC curve (AUC), classification performance is shown for models considering **(A)** 2 groups (CD vs. HC, UC vs. HC, CD vs. UC, CD vs. IC, UC vs. IC, IC vs. HC), **(B)** 3 groups (CD vs. UC+HC, UC vs. CD+HC, HC vs. CD+UC, CD vs. UC+IC, UC vs. CD+IC, IC vs. CD+UC) and **(C)** 4 groups (CD vs. UC+HC+IC, UC vs. CD+HC+IC, HC vs. CD+UC+IC, IC vs. CD+UC+HC). Performance of linear standard SVMs (considering every miRNA measured, white boxes) is compared to linear elastic SCAD SVMs (considering subsets of miRNAs measured, red boxes). In addition, as a measure of model complexity the percentage of miRNAs neglected for constructing the respective penalized SVMs are plotted (blue boxes).

**Fig 1**summarizes the elastic SCAD SVM’s performance in solving the 16 different diagnostic problems measured by the area under the curve (AUC). The models incorporating 2 groups show stable superiority (median AUC = 0.97; 0.98 including vs. 0.95 excluding IC) in comparison to the models considering 3 groups (median AUC = 0.92; 0.93 including vs. 0.92 excluding IC) or 4 groups (median AUC = 0.85). In addition, these models provide remarkable sparsity (median percentage of miRNAs removed = 99.3%, 99.4% including vs. 99.2% excluding IC) and only marginal loss of performance compared to the standard SVM. As shown in **Table 2**, in terms of median sensitivity and specificity, the performance of the selected models can be estimated as 1.00 and 0.90, respectively (1.00 and 0.91 including IC, 1.00 and 0.90 excluding IC). The median balanced accuracy (BAC) was 0.95 (0.96 including IC, 0.95 excluding IC). Additional performance measures (e.g. median Matthews correlation coefficient (MCC) and Youden’s index (YOUDEN)) are listed in **S3 Table**for each particular classifier.

**Table 2:** Performance measures for the different classification models. Corresponding to the classification accuracy of the sparsest median performing penalized SVM (see **Fig 1**) for each classifier area under the ROC curve (AUC), sensitivity (SN = TPR, true positive rate), specificity (SP = TNR, true negative rate) and balanced accuracy (BAC = (SN+SP)/2) are shown.

#groups	classifier	AUC	SN	SP	BAC
	CD/HC	0.950	0.963	1.000	0.981
	UC/HC	0.981	1.000	0.900	0.950
	CD/UC	0.889	1.000	0.833	0.917
2	**median**	**0.950**	**1.000**	**0.900**	**0.950**
	CD/IC	0.984	1.000	0.909	0.955
	UC/IC	1.000	1.000	1.000	1.000
	IC/HC	0.752	0.750	0.765	0.757
	**median**	**0.984**	**1.000**	**0.909**	**0.955**
	CD/UC+HC	0.893	0.969	0.692	0.831
	UC/CD+HC	0.917	0.971	0.800	0.886
	HC/CD+UC	0.974	1.000	0.963	0.981
3	**median**	**0.917**	**0.971**	**0.800**	**0.886**
	CD/UC+IC	0.876	0.951	0.800	0.876
	UC/CD+IC	0.933	0.976	0.889	0.933
	IC/CD+UC	0.984	0.950	1.000	0.975
	**median**	**0.933**	**0.951**	**0.889**	**0.933**
	CD/UC+HC+IC	0.885	0.970	0.800	0.885
	UC/CD+HC+IC	0.930	0.985	0.800	0.893
4	HC/CD+UC+IC	0.758	0.830	0.708	0.769
	IC/CD+UC+HC	0.817	0.860	0.765	0.813
	**median**	**0.851**	**0.915**	**0.783**	**0.849**

**Fig 2:**
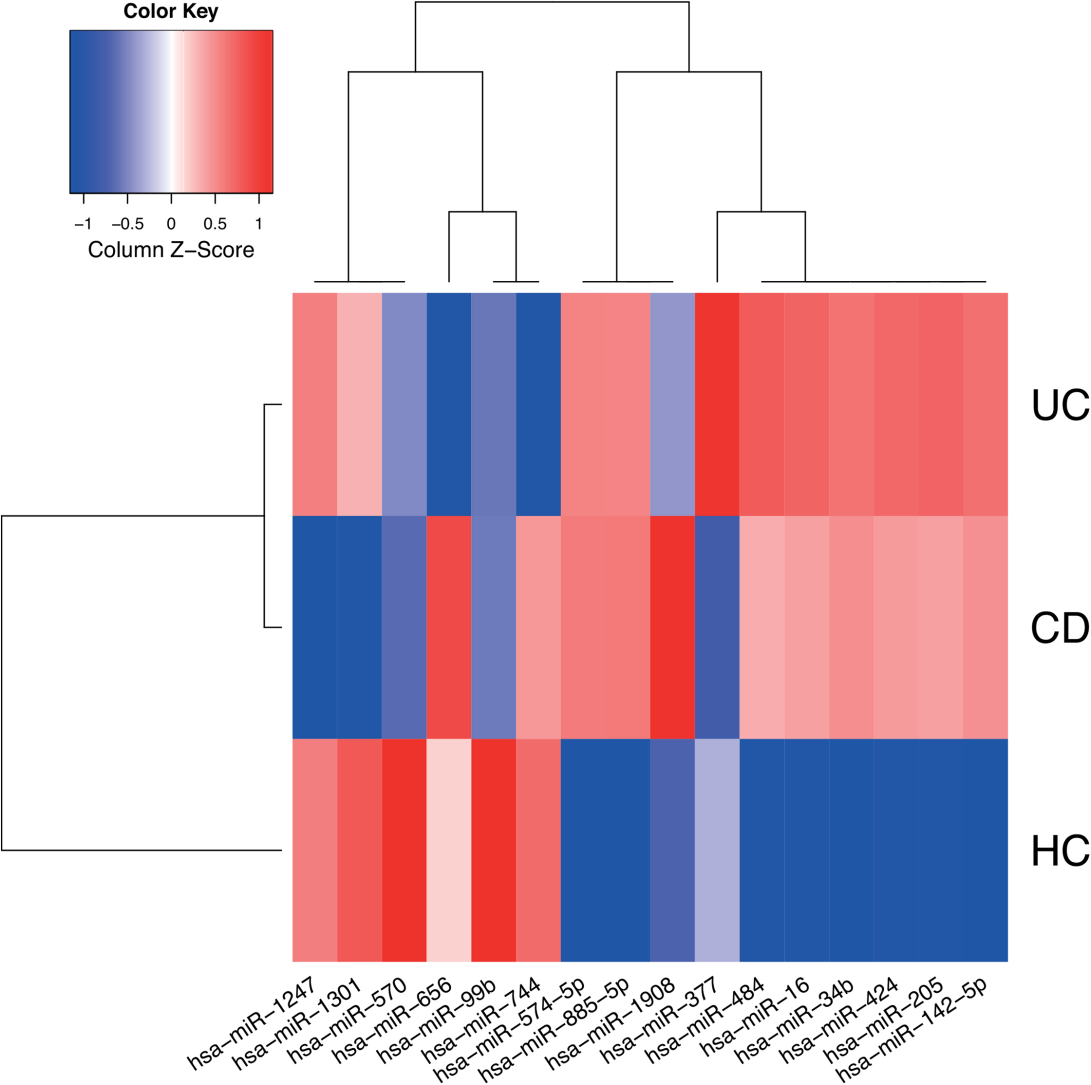
Expression profile of signature miRNAs. Median normalized expression levels for miRNAs considered by the final models (sparsest median performing elastic SCAD SVMs) used to distinguish CD, UC and HC are shown. The heat map was generated using a distance function based on Spearman’s rank correlation coefficient and agglomerative hierarchical clustering using complete-linkage. Low and high expression levels are plotted using red and blue, respectively.

The final set of markers selected for diagnostic application is shown in **Fig 2**. It includes 16 distinct miRNAs originating from elastic SCAD SVMs incorporating 2 groups: hsa-miR-34b-3p, hsa-miR-142-5p, hsa-miR-205-5p, hsa-miR-424-5p, hsa-miR-570-3p, hsa-miR-885-5p, hsa-miR-1301-3p (CD vs. HC), hsa-miR-16-5p, hsa-miR-34b-3p, hsa-miR-99b-5p (UC vs. HC) and hsa-miR-34b-3p, hsa-miR-377-3p, hsa-miR-484, hsa-miR-574-5p, hsa-miR-656-3p, hsa-miR-744-5p, hsa-miR-1247-5p, hsa-miR-1908-5p (CD vs. UC, miRBase version 21 nomenclature). The corresponding models provide tools for the distinction of CD and UC as well as CD, UC and HC with remarkable small classification error rates of 3.1 and 3.3%, respectively (i.e. applying the proposed models will result in approximately 3 incorrect diagnoses per 100 tests). Notably, these estimates are not based on an independent dataset. Therefore, they are potentially optimistic but still provide a measure for the combined classifier’s diagnostic value. This is confirmed by permutation tests showing significant deviation of the classification error from its random expectation. For further examples of classifier combinations and the corresponding size of miRNA signatures see S6 **Table**.

### Validation using random forests

A second, independent machine learning approach, random forest analysis (RFs), was employed to validate our SVM-based miRNA signatures. As shown in **Table 3**, random forest analyses confirmed our SVM results. In the case of models considering 2 groups, the performance differences were small (AUC = 0.990 for the SVM using the entirety of the miRNAs, AUC = 0.992 for the RF per holdout sample using miRNAs selected by the RF and AUC = 0.996 for the RF using the top 50% of the miRNAs most frequently selected by the RF across all runs). When using the miRNAs selected by the elastic SCAD SVM for training the RF in the same way, highly accurate models were obtained: AUC = 0.965 for the elastic SCAD SVM, AUC = 0.992 for the RF per holdout sample using miRNAs selected by the elastic SCAD SVM and AUC = 0.994 for the RF using the miRNAs considered by the median performing elastic SCAD SVM. These results strongly support the validity of the miRNA combinations chosen as putative diagnostic markers by the SVM approach.

**Table 3:** Comparison of classification approaches. The table shows the median classifier performance (AUC) of the classification problems considering different numbers of groups (2, 3 and 4) and models (SVM and RF). Performance of the standard SVM is compared to the elastic SCAD SVM (a). Performance of the RF per holdout sample using the miRNAs selected by the RF is compared to the RF per holdout sample using the miRNAs selected by the elastic SCAD SVM (b). Performance of the RF using the top 50% of the miRNAs most frequently selected by the RF across all runs is compared to the RF using the miRNAs selected by the median performing elastic SCAD SVM (c). For each comparison performance estimates based on miRNAs selected by the elastic SCAD SVM are enclosed in parentheses.

classification signature	2 groups	2 groups, no IC	3 groups	3 groups, no IC	4 groups
(a) SVM	0.990 (0.965)	0.981 (0.950)	0.938 (0.925)	0.931 (0.917)	0.858 (0.851)
(b) RF, holdout signature	0.992 (0.992)	0.985 (0.985)	0.978 (0.980)	0.969 (0.974)	0.919 (0.910)
(c) RF, top/median signature	0.996 (0.994)	0.992 (0.988)	0.982 (0.989)	0.977 (0.992)	0.941 (0.940)

### Target genes of the diagnostic miRNA signature correlate with susceptibility genes

To assess the potential biological significance of miRNAs within the signatures revealed by the machine-learning approaches we correlated previous knowledge about disease relevance of IBD-related genes to the experimentally validated target genes of the miRNA signatures (results summarized in **Table 4**and **S7 Table**). Irrespective of the miRNA signature tested (CD vs. HC, UC vs. HC or CD vs. UC), we observed an overlap of miRNA target genes and published IBD-related genes or, on the other hand, genes within known IBD susceptibility loci. Thus, target genes of signature miRNAs used for the distinction of CD and HC are significantly enriched for loci known to be associated with CD (p = 3.22∙10^−3^), UC (p = 1.09∙10^−3^) and suggestive for IBD (p = 4.37∙10^−2^). Targets of signature miRNAs used for the distinction of UC and HC show suggestive enrichment for loci associated with CD (p = 3.34∙10^−2^). Considering the targets of the complete set of miRNAs used for the distinction of CD, UC and HC suggestive enrichment is observed for previously published susceptibility loci of CD (p = 4.80∙10^−3^) and UC (p = 4.80∙10^−3^).

**Table 4:** Signature miRNAs regulate target genes previously identified as IBD-risk genes. Both CD and UC diagnostic signatures contain several miRNAs that regulate experimentally validated target genes known to be involved in IBD-related phenotypes in humans and/or mice. Genes marked with * have even been reported as candidate genes in susceptibility loci identified in recent IBD GWAS.

signature	miRNA	target gene	function/disease implication	reference
CD/HC	hsa-miR-205	LRRK2 *	susceptibility gene for CD	[63]
		SHIP2/INPPL1	regulator of PI3K, therapeutical target in inflammation	[64]
		ZEB1	regulates intestinal cell growth	[65]
		E2F1	activation promoted by chronic inflammation	[66]
		ERBB3	inhibits treatment of IBD	[67]
	hsa-miR-142-5p	NFE2L2/NRF2	susceptibility for DSS-induced colitis	[68]
	hsa-miR-424	MYB	colonic epithelial disruption by mir-150	[69]
		CUL2 *	susceptibility gene for CD	[70]
		PU.1	role in T-cell mediated colitis	[71]
	hsa-miR-34b	HNF4A *	susceptibility gene for early onset CD	[72]
		CREB1	diverse implications in CD	[73]
UC/HC	hsa-miR-34b	HNF4A *	susceptibility gene for UC	[74]
		NOTCH1	regulator of intestinal epithelial barrier	[75]
		c-MET/HGFR	upregulated in UC	[76]
		CAV1	upregulated in UC inflamed tissue	[77]
	hsa-miR-99b	RAVER2 *	susceptibility gene for UC	[78]
		mTOR	inhibition depletes mouse colitis	[79]
	hsa-miR-16	HMGA1/2	P-ANCA autoantigens	[80]
		ACVR2a	associated with IBD-related CRC	[81]

In a next step we investigated whether previously identified genetic variation in the IBD susceptibility genes could directly play a role in miRNA-target gene interaction. We used dbSNP annotations of the human genome provided by the UCSC genome browser to identify SNPs that could interfere with miRNA binding sites. As a result we found that most 3’-UTRs of the analyzed IBD-risk genes indeed exhibit genetic variation (SNPs and small InDels) but mostly not in the respective signature-miRNA binding site regions. Only for hsa-miR-99b, which is part of the UC signature, we were able to identify potentially interesting SNPs located in the essential miRNA binding site seed regions of RAVER2 (rs183861354, chr1:64831085, G>A) and mTOR (rs375505566, chr1:11107188, G>A). Strikingly, both SNPs change the same nucleotide position within the seed region of the miRNA binding site. Whether this single nucleotide variant affects the binding behavior and as a consequence the gene functions in IBD cases compared to healthy controls, remains to be shown.

## Discussion

In this study we compared miRNA expression profiles of whole peripheral blood samples from patients with inflammatory bowel disease (Crohn’s disease and ulcerative colitis) to healthy controls and “disease controls”. We were able to confirm significantly deregulated miRNAs in blood that were previously reported by others and could further add new candidates to the catalogue of IBD-associated miRNAs. To our knowledge this study represents the largest (both in terms of samples and measured miRNAs) blood-based miRNA-expression study for IBD published to date. Our analysis, however, was focused on the identification of disease specific, diagnostic classification signatures derived from the overall miRNA expression profiles irrespective of single miRNA deregulation.

miRNAs are often referred to as “blood-based biomarkers” for diagnosing disease or monitoring disease progression. As it has been shown for several types of cancer this holds true as long as a relatively stable condition, such as a recurrent aberrant gene expression in certain tissues or exosomal miRNA content can be measured repeatedly. Concerning blood-based miRNA expression in inflammatory or auto-immune diseases, however, the assumption of stable conditions is often violated. Numerous known comorbidities as well as environmental and life-style factors, treatment and disease activity may influence miRNA levels in the blood stream and lead to intra- and interindividual miRNA-expression variability. Also general factors like blood cell composition, depending on the type of disease may vary significantly, and hence impact miRNA levels in peripheral blood. Thus, instead of aiming to identify single miRNA “biomarkers”, to enhance predictive power it appears more promising to investigate complex predictors that are based on larger numbers of miRNAs. In this way, besides simple deregulation, also certain combinations of regulatory effects are taken into account for diagnostic or predictive models.

In this work we demonstrated the use of machine-learning techniques to construct IBD-specific miRNA signatures and we were able to reveal highly accurate classification models that distinguish healthy and diseased individuals as well as the two main IBD subtypes and other inflammatory conditions from each other. Furthermore, a minimal set of not more than 16 miRNAs, being sufficient for sensitive and specific classification, holds great promises and should be further evaluated in independent sample panels.

The here-investigated models represent solutions to construct classifiers for miRNA expression data but they also exhibit some limitations, most notably the limited generalizability of the models to other technologies. All models are trained based on the same type of data that originate from a certain technology (here the Geniom Array). Application of these models to independent samples in a clinical or diagnostic setting would always require to remove technology biases. In addition to that, the here-presented classifiers remain restricted to the set of miRNAs that are present on the microarray used to detect differential expression. Future studies utilizing next generation sequencing (NGS) will presumably overcome this limitation as all present miRNAs in a sample are theoretically detectable by this technology. Furthermore, implementing approaches that include more levels of available information e.g. genetic variants, microbiome data or clinical data from electronic health records (that include information on differential diagnoses, medication, disease activity, etc.) will potentially add to the predictive power needed for highly sensitive and specific classification.

Regularized instances of support vector machines incorporate penalties for model complexity to prevent overfitting and to provide sparse solutions. In the here-presented study this property is used to obtain small sets of miRNAs suitable for diagnostic application. It is expected that miRNAs essential for solving a particular classification problem likewise are selected by random forests using the recurrent relative variable importance. However, this approach does not aim at selecting a minimal set of features so that one does not expect miRNA signatures to be fully overlapping. To obtain more comparable results, future studies might consider regularized random forests as introduced by Deng and Runger [82]. In this work the miRNA signature selected using the elastic SCAD SVM was confirmed by comparably high classification performance of random forests as an independent classification approach. For this purpose afore-mentioned limitations can be neglected.

To obtain a model applicable with high accuracy to independent data we chose the sparsest median performing elastic SCAD SVM along with the corresponding miRNA signature. Both, the regularization approach and the comprehensive holdout sampling decrease the model’s probability of being overfitted to the dataset generated for this study. However, due to correlating expression profiles it is expected that models with matching accuracy potentially incorporate differing miRNAs. For the same reason more complex signatures may exist which merely incoporate additional highly correlated miRNAs.

Classifiers for complex diagnostic problems were constructed by majority voting of simpler models. As shown in this study, this approach results in remarkable low classification error rates. However, follow-up studies could potentially incorporate the estimation of class probabilities to enhance the interpretability of the classification results.

To get insights into functional implications of the miRNAs contained in the revealed IBD signature, we screened current databases for experimentally validated miRNA-target gene interactions. Notably, a considerable fraction of the target genes within the IBD miRNA signatures has been implicated in intestinal diseases (see **Table 4**). Many of those targets were identified in recent IBD GWAS but most of the genetic variation detected does not correlate (and thus not interfere) with miRNA regulatory binding sites. Only the hsa-mir-99b binding sites in the 3’-UTRs of the IBD susceptibility gene RAVER2, a ribonucleoprotein (hnRNP) involved in regulation of splicing and mTOR, a serine/threonine proteine kinase, shown to be involved in activation of autophagy, represent good candidates for further experimental investigation. In the future, more complete data on genetic varation in 3’-UTRs of IBD related genes will supposedly come from whole genome sequencing approaches and will thus enable for more complete analyses of miRNA target genes. In a recent review on genetic studies in IBD Liu and Anderson [83] conclude that most of the identified GWAS loci actually reside in noncoding regions of the genome and that a vast number of these noncoding variants will likely play a role in gene regulation. miRNAs are certainly an important part of the regulatory machinery of the genome, but besides their utility in diagnostics, miRNA signatures might also give valuable insights into disease development and progression.

## Supporting Information

S1 Fig MDS (multidimensional scaling) plots for visualization of background-subtracted intensity values. Background-subtracted intensity values normalized using variance stabilization **(A)** before and **(B)** after median centering based on the batches observed for healthy controls. The corresponding medians are indicated by black circles. MDS was performed using a distance function based on Spearman’s rank correlation coefficient. Data points of each group are represented by their α-shape (generalized convex hull). The second plot visualizes the batch-corrected normalized data used for diagnostic classification.
(TIFF)

S2 Fig Median expression levels of miRNAs previously published as being deregulated in CD, UC and HC. The horizontal side bar indicates the correspondence between the literature and the dataset used for this study. Measurements with directions of effect deviating from the literature are marked using black bars. The heat map was generated using a distance function based on Spearman’s rank correlation coefficient and agglomerative hierarchical clustering using complete-linkage. Low and high expression levels are plotted using red and blue, respectively.
(TIFF)

S3 Fig Median expression profiles of significantly deregulated miRNAs in CD, UC and HC. For each pair of groups two-sample t-tests were applied. Deregulation was considered as being significant for Holm-corrected p-values <0.05. Not significantly differentially expressed miRNAs were neglected. 667 out of 863 miRNAs were differentially deregulated in any of the comparisons.
(TIFF)

S4 Fig Classification results for LASSO SVM. Measured by the area under the ROC curve (AUC) classification performance is shown for models considering **(A)** 2 groups (CD vs. HC, UC vs. HC, CD vs. UC, CD vs. IC, UC vs. IC, IC vs. HC), **(B)** 3 groups (CD vs. UC+HC, UC vs. CD+HC, HC vs. CD+UC, CD vs. UC+IC, UC vs. CD+IC, IC vs. CD+UC) and **(C)** 4 groups (CD vs. UC+HC+IC, UC vs. CD+HC+IC, HC vs. CD+UC+IC, IC vs. CD+UC+HC). Performance of linear standard SVMs (considering every miRNA measured, white boxes) is compared to linear LASSO SVMs (considering subsets of miRNAs measured, red boxes). In addition, as a measure of model complexity the percentage of miRNAs neglected for constructing the respective penalized SVMs are plotted (blue boxes).
(TIFF)

S5 Fig Classification results for elastic net SVM. Measured by the area under the ROC curve (AUC) classification performance is shown for models considering **(A)** 2 groups (CD vs. HC, UC vs. HC, CD vs. UC, CD vs. IC, UC vs. IC, IC vs. HC), **(B)** 3 groups (CD vs. UC+HC, UC vs. CD+HC, HC vs. CD+UC, CD vs. UC+IC, UC vs. CD+IC, IC vs. CD+UC) and **(C)** 4 groups (CD vs. UC+HC+IC, UC vs. CD+HC+IC, HC vs. CD+UC+IC, IC vs. CD+UC+HC). Performance of linear standard SVMs (considering every miRNA measured, white boxes) is compared to linear elastic net SVMs (considering subsets of miRNAs measured, red boxes). In addition, as a measure of model complexity the percentage of miRNAs neglected for constructing the respective penalized SVMs are plotted (blue boxes).
(TIFF)

S6 Fig Classification results for SCAD SVM. Measured by the area under the ROC curve (AUC) classification performance is shown for models considering **(A)** 2 groups (CD vs. HC, UC vs. HC, CD vs. UC, CD vs. IC, UC vs. IC, IC vs. HC), **(B)** 3 groups (CD vs. UC+HC, UC vs. CD+HC, HC vs. CD+UC, CD vs. UC+IC, UC vs. CD+IC, IC vs. CD+UC) and **(C)** 4 groups (CD vs. UC+HC+IC, UC vs. CD+HC+IC, HC vs. CD+UC+IC, IC vs. CD+UC+HC). Performance of linear standard SVMs (considering every miRNA measured, white boxes) is compared to linear SCAD SVMs (considering subsets of miRNAs measured, red boxes). In addition, as a measure of model complexity the percentage of miRNAs neglected for constructing the respective penalized SVMs are plotted (blue boxes).
(TIFF)

S7 Fig Comparison of SVM and random forest. Measured by the area under the ROC curve (AUC) classification performance is shown for models considering **(A)** 2 groups (CD vs. HC, UC vs. HC, CD vs. UC, CD vs. IC, UC vs. IC, IC vs. HC), **(B)** 3 groups (CD vs. UC+HC, UC vs. CD+HC, HC vs. CD+UC, CD vs. UC+IC, UC vs. CD+IC, IC vs. CD+UC) and **(C)** 4 groups (CD vs. UC+HC+IC, UC vs. CD+HC+IC, HC vs. CD+UC+IC, IC vs. CD+UC+HC). Classification performance of the linear elastic SCAD SVM (white box) is compared to a **Random forests per holdout sample considering variables selected using the SVM** (red box) and the Random forest itself (blue box), respectively.
(TIFF)

S8 Fig Comparison of SVM and random forest. Measured by the area under the ROC curve (AUC) classification performance is shown for models considering **(A)** 2 groups (CD vs. HC, UC vs. HC, CD vs. UC, CD vs. IC, UC vs. IC, IC vs. HC), **(B)** 3 groups (CD vs. UC+HC, UC vs. CD+HC, HC vs. CD+UC, CD vs. UC+IC, UC vs. CD+IC, IC vs. CD+UC) and **(C)** 4 groups (CD vs. UC+HC+IC, UC vs. CD+HC+IC, HC vs. CD+UC+IC, IC vs. CD+UC+HC). Classification performance of the linear elastic SCAD SVM (white box) is compared to a **Random forests considering variables selected using the median performing SVM** (red box). Additionally, Random forests were trained with the top 50% of the variables ranked by their frequency of selection (blue box).
(TIFF)

S1 Table Differential expression analysis. For each binary combination of groups t-tests for differential miRNA expression were conducted. The test results are summarized by the fold change (fc), the t-statistic (t), the p-value (p) and the p-values adjusted for multiple testing using Holm-correction (padj).
(XLSX)

S2 Table miRNAs previously described to be deregulated in IBD. Tables were adapted from Chen *et al*. **(A)** and Coscun et al. **(B)**, respectively. For binary comparisons (CD vs. HC, UC vs. HC, IBD vs. HC and CD vs. UC). Directions of effect known from the literature as well as measured by the microarray used for this study are summarized.
(XLSX)

S3 Table Performance measures for LASSO SVM. Corresponding to the classification accuracy of the sparsest median performing penalized SVM (see S4 Fig) for each classification task area under the ROC curve (AUC), Matthews correlation coefficient (MCC), balanced accuracy (BAC), Youden’s index (YOUDEN), sensitivity (SN = TPR), specificity (SP = TNR), positive predictive value (PPV), false discovery rate (FDR), negative predictive value (NPV) and false omission rate (FOR) are shown.
(XLSX)

S4 Table Performance measures for elastic net SVM. Corresponding to the classification accuracy of the sparsest median performing penalized SVM (see **S5 Fig**) for each classification task area under the ROC curve (AUC), Matthews correlation coefficient (MCC), balanced accuracy (BAC), Youden’s index (YOUDEN), sensitivity (SN = TPR), specificity (SP = TNR), positive predictive value (PPV), false discovery rate (FDR), negative predictive value (NPV) and false omission rate (FOR) are shown.
(XLSX)

S5 Table Performance measures for SCAD SVM. Corresponding to the classification accuracy of the sparsest median performing penalized SVM (see **S6 Fig**) for each classification task area under the ROC curve (AUC), Matthews correlation coefficient (MCC), balanced accuracy (BAC), Youden’s index (YOUDEN), sensitivity (SN = TPR), specificity (SP = TNR), positive predictive value (PPV), false discovery rate (FDR), negative predictive value (NPV) and false omission rate (FOR) are shown.
(XLSX)

S6 Table Exemplary diagnostic application. The final median performing models were used to predict the disease status based on each individual’s miRNA expression data. For each combined classifier, constructed using majority voting, the number of groups, n(groups), considered by the atomic models as well as the respective number of miRNAs, n(mirnas), and unique miRNAs, n(unique), are shown. In addition the classification errors per individual’s group (ε(CD), ε(UC), ε(HC) and ε(IC)) were estimated. Furthermore, for each classifier the mean classification error (mean) as well as the corresponding permutation based Z-score (Z(mean)) were calculated. Z-scores corresponding to p-values lower than the significance threshold of 0.05 are marked using *.
(XLSX)

S7 Table Enrichment analysis for validated signature miRNA targets. The proportion of validated targets of the miRNAs selected for diagnostic prediction (CD vs. HC, UC vs. HC, CD vs. UC and CD vs. UC vs. HC) known to be coded at risk loci (CD, UC and IBD) is compared to the proportion of general miRNA targets known to be coded at risk loci (CD, UC and IBD). Targets of signature miRNA coded at risk and non-risk loci are denoted as C_r and C, repectively. miRNA targets excluding signature targets coded at risk and non-risk loci are denoted as R_r and R, repectively. The total number of validated miRNA targets is denoted as N. Enrichment analysis was performed using Fisher’s exact test, resulting p-values are marked as being nominal significant (*, p<0.05) and significant after Bonferroni correction for multiple testing (**; padj<0.05).
(XLSX)

S8 Table Raw miRNA expression data. Background subtracted microarray intensities from Geniom Wizard Software that were used to infer miRNA expression levels.
(XLSX)
