# Supplementary figures and images for: Sparse Modeling Reveals miRNA Signatures for Diagnostics of Inflammatory Bowel Disease
Source: PLoS One. 2015 Oct 14;10(10):e0140155. doi: 10.1371/journal.pone.0140155 (PMC4605644; doi:10.1371/journal.pone.0140155)

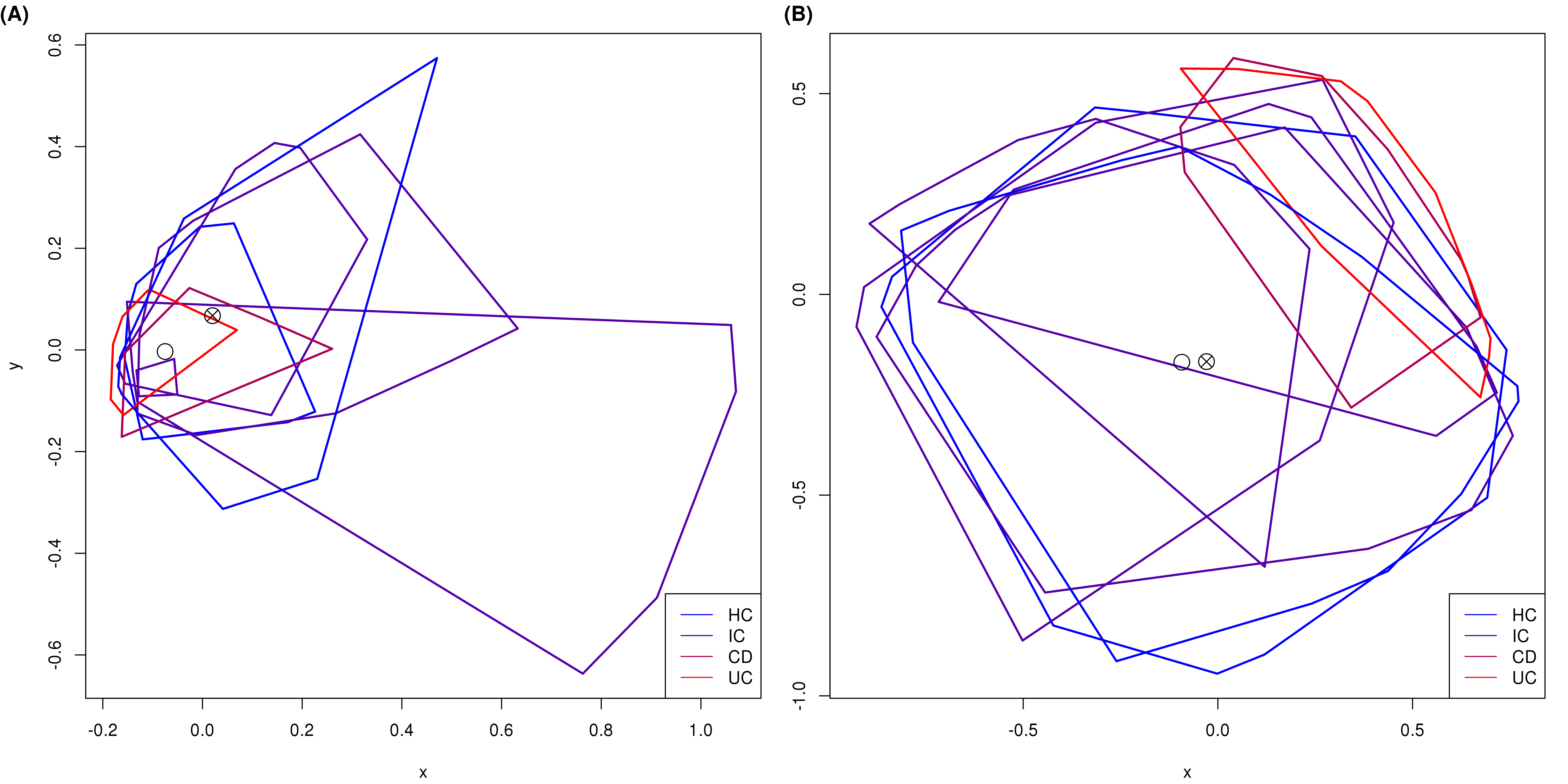

Supplement: S1 Fig — Background-subtracted intensity values normalized using variance stabilization (A) before and (B) after median centering based on the batches observed for healthy controls. The corresponding medians are indicated by black circles. MDS was performed using a distance function based on Spearman’s rank correlation coefficient. Data points of each group are represented by their α-shape (generalized convex hull). The second plot visualizes the batch-corrected normalized data used for diagnostic classification. (TIFF) [file pone.0140155.s001.tiff]

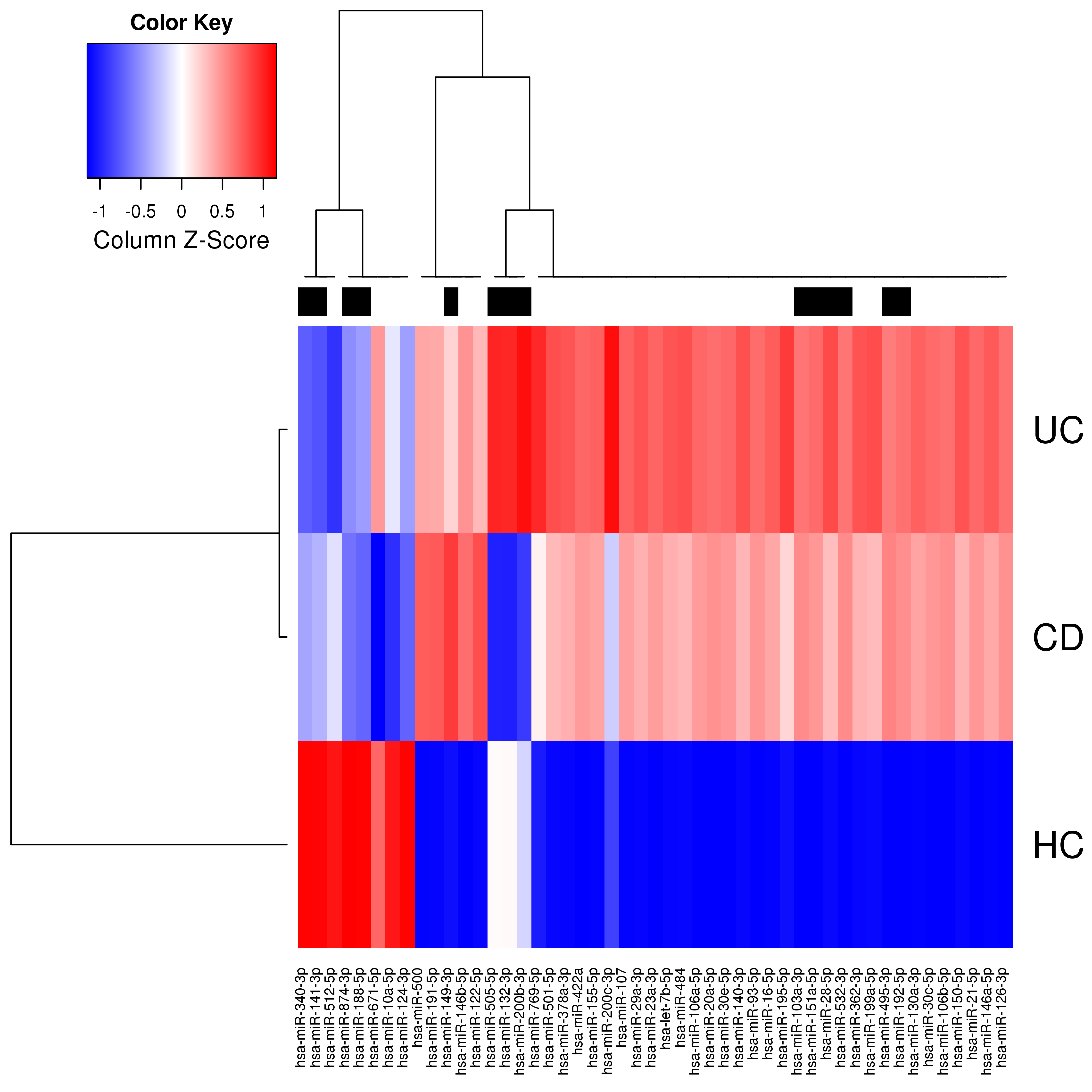

Supplement: S2 Fig — The horizontal side bar indicates the correspondence between the literature and the dataset used for this study. Measurements with directions of effect deviating from the literature are marked using black bars. The heat map was generated using a distance function based on Spearman’s rank correlation coefficient and agglomerative hierarchical clustering using complete-linkage. Low and high expression levels are plotted using red and blue, respectively. (TIFF) [file pone.0140155.s002.tiff]

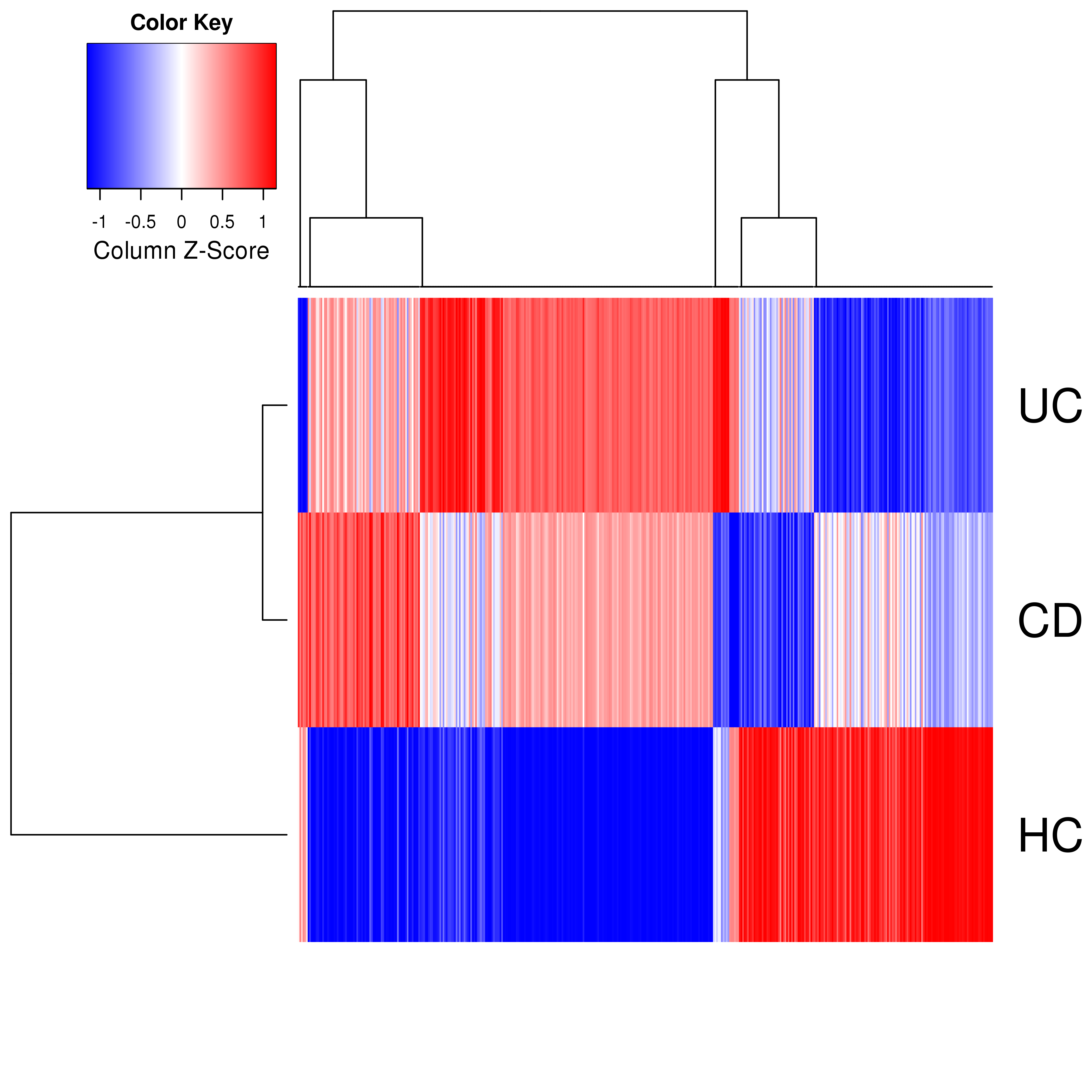

Supplement: S3 Fig — For each pair of groups two-sample t-tests were applied. Deregulation was considered as being significant for Holm-corrected p-values <0.05. Not significantly differentially expressed miRNAs were neglected. 667 out of 863 miRNAs were differentially deregulated in any of the comparisons. (TIFF) [file pone.0140155.s003.tiff]

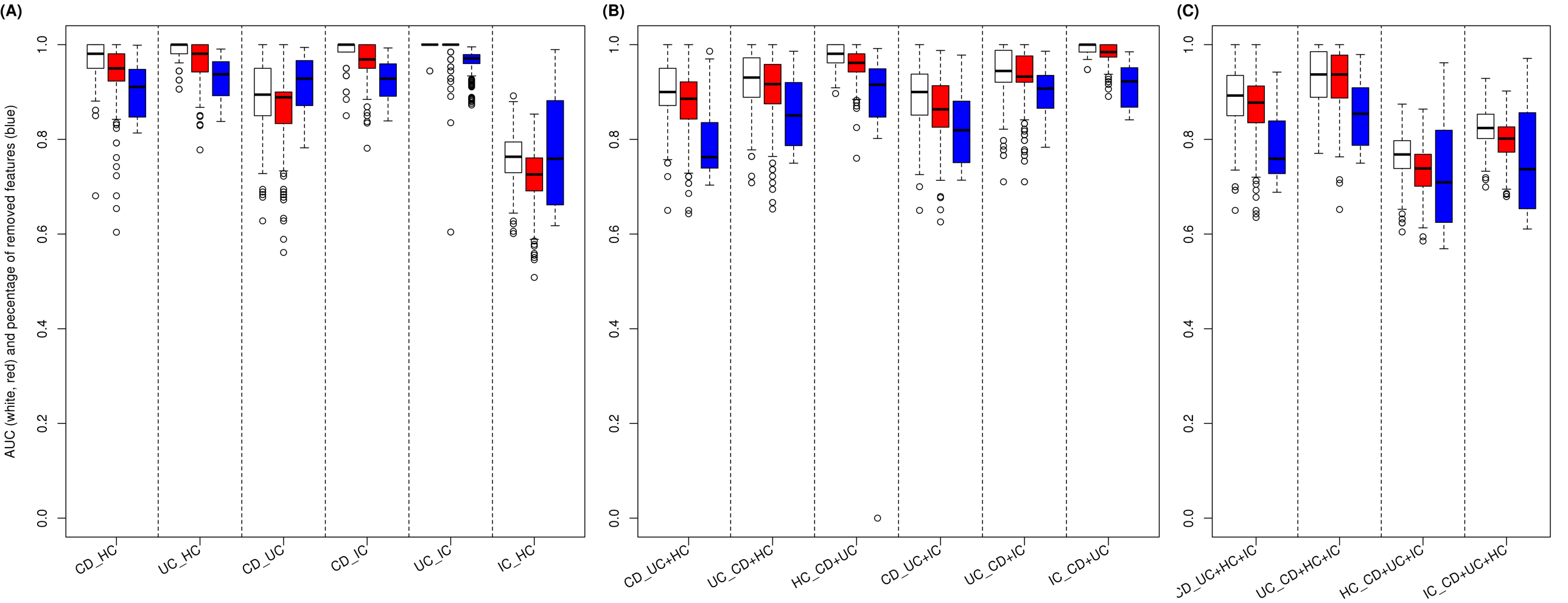

Supplement: S4 Fig — Measured by the area under the ROC curve (AUC) classification performance is shown for models considering (A) 2 groups (CD vs. HC, UC vs. HC, CD vs. UC, CD vs. IC, UC vs. IC, IC vs. HC), (B) 3 groups (CD vs. UC+HC, UC vs. CD+HC, HC vs. CD+UC, CD vs. UC+IC, UC vs. CD+IC, IC vs. CD+UC) and (C) 4 groups (CD vs. UC+HC+IC, UC vs. CD+HC+IC, HC vs. CD+UC+IC, IC vs. CD+UC+HC). Performance of linear standard SVMs (considering every miRNA measured, white boxes) is compared to linear LASSO SVMs (considering subsets of miRNAs measured, red boxes). In addition, as a measure of model complexity the percentage of miRNAs neglected for constructing the respective penalized SVMs are plotted (blue boxes). (TIFF) [file pone.0140155.s004.tiff]

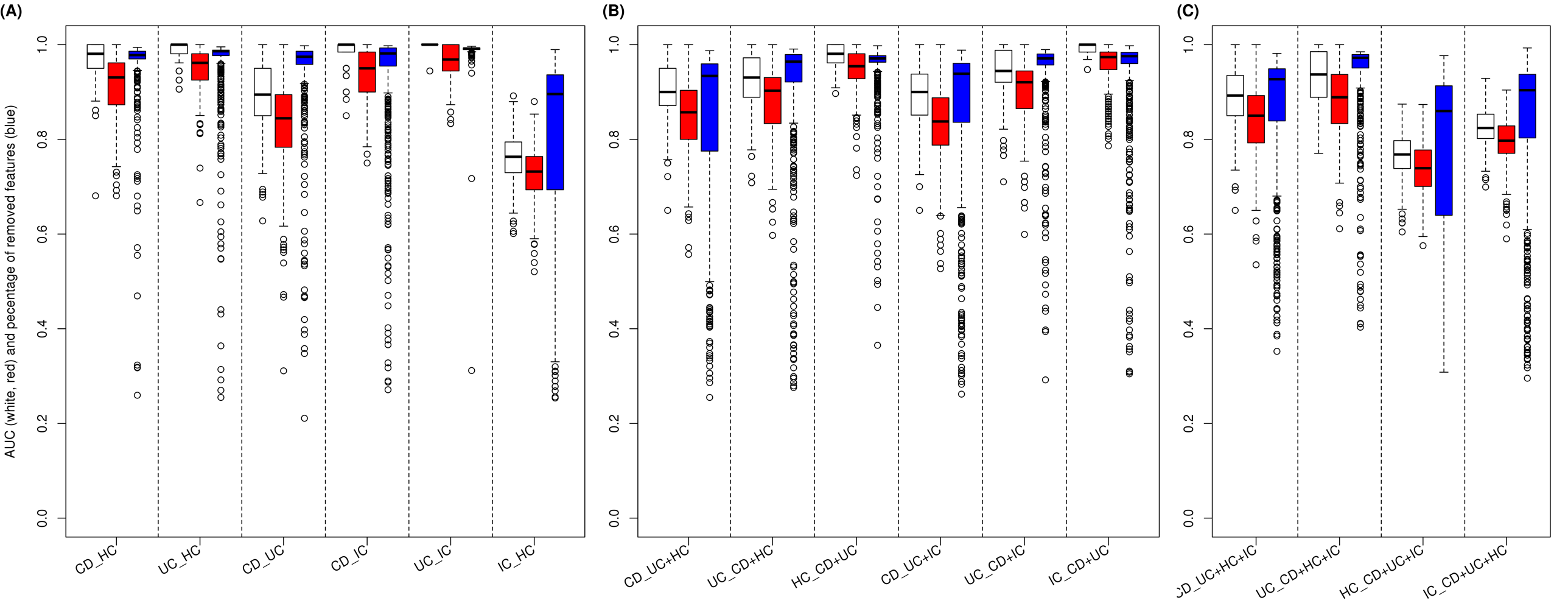

Supplement: S5 Fig — Measured by the area under the ROC curve (AUC) classification performance is shown for models considering (A) 2 groups (CD vs. HC, UC vs. HC, CD vs. UC, CD vs. IC, UC vs. IC, IC vs. HC), (B) 3 groups (CD vs. UC+HC, UC vs. CD+HC, HC vs. CD+UC, CD vs. UC+IC, UC vs. CD+IC, IC vs. CD+UC) and (C) 4 groups (CD vs. UC+HC+IC, UC vs. CD+HC+IC, HC vs. CD+UC+IC, IC vs. CD+UC+HC). Performance of linear standard SVMs (considering every miRNA measured, white boxes) is compared to linear elastic net SVMs (considering subsets of miRNAs measured, red boxes). In addition, as a measure of model complexity the percentage of miRNAs neglected for constructing the respective penalized SVMs are plotted (blue boxes). (TIFF) [file pone.0140155.s005.tiff]

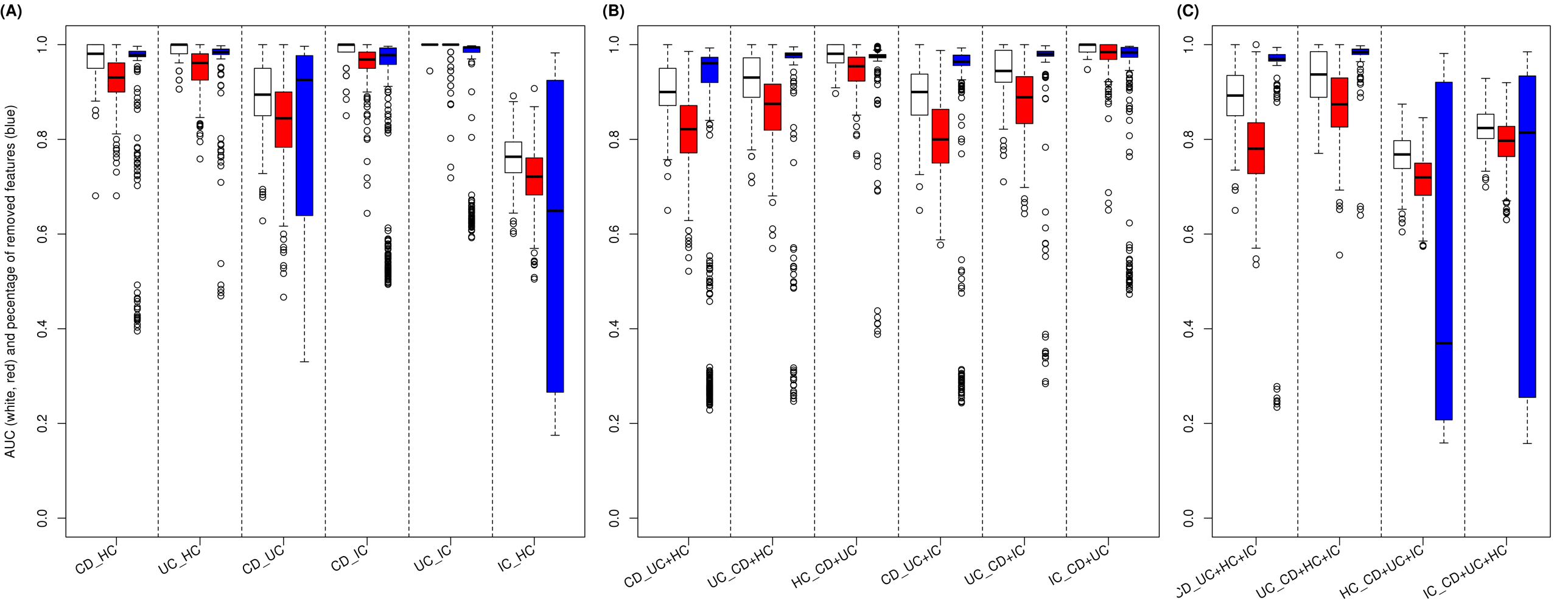

Supplement: S6 Fig — Measured by the area under the ROC curve (AUC) classification performance is shown for models considering (A) 2 groups (CD vs. HC, UC vs. HC, CD vs. UC, CD vs. IC, UC vs. IC, IC vs. HC), (B) 3 groups (CD vs. UC+HC, UC vs. CD+HC, HC vs. CD+UC, CD vs. UC+IC, UC vs. CD+IC, IC vs. CD+UC) and (C) 4 groups (CD vs. UC+HC+IC, UC vs. CD+HC+IC, HC vs. CD+UC+IC, IC vs. CD+UC+HC). Performance of linear standard SVMs (considering every miRNA measured, white boxes) is compared to linear SCAD SVMs (considering subsets of miRNAs measured, red boxes). In addition, as a measure of model complexity the percentage of miRNAs neglected for constructing the respective penalized SVMs are plotted (blue boxes). (TIFF) [file pone.0140155.s006.tiff]

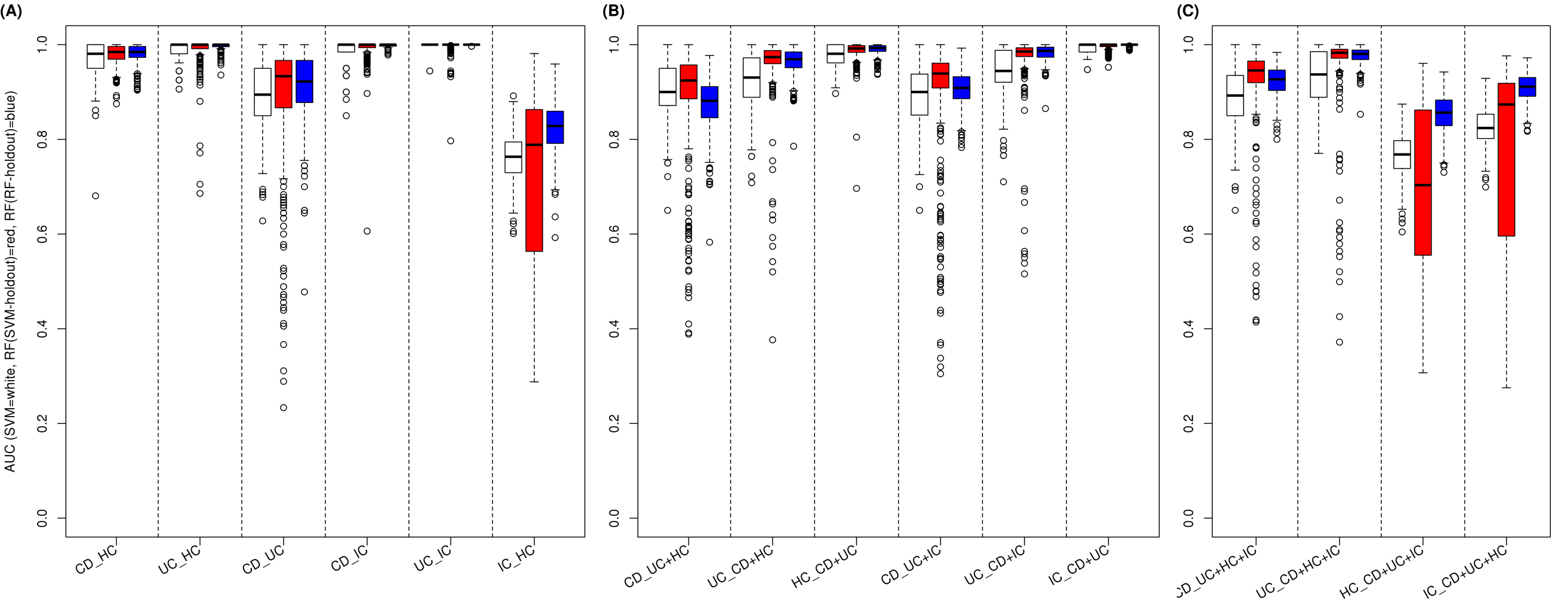

Supplement: S7 Fig — Measured by the area under the ROC curve (AUC) classification performance is shown for models considering (A) 2 groups (CD vs. HC, UC vs. HC, CD vs. UC, CD vs. IC, UC vs. IC, IC vs. HC), (B) 3 groups (CD vs. UC+HC, UC vs. CD+HC, HC vs. CD+UC, CD vs. UC+IC, UC vs. CD+IC, IC vs. CD+UC) and (C) 4 groups (CD vs. UC+HC+IC, UC vs. CD+HC+IC, HC vs. CD+UC+IC, IC vs. CD+UC+HC). Classification performance of the linear elastic SCAD SVM (white box) is compared to a Random forests per holdout sample considering variables selected using the SVM (red box) and the Random forest itself (blue box), respectively. (TIFF) [file pone.0140155.s007.tiff]

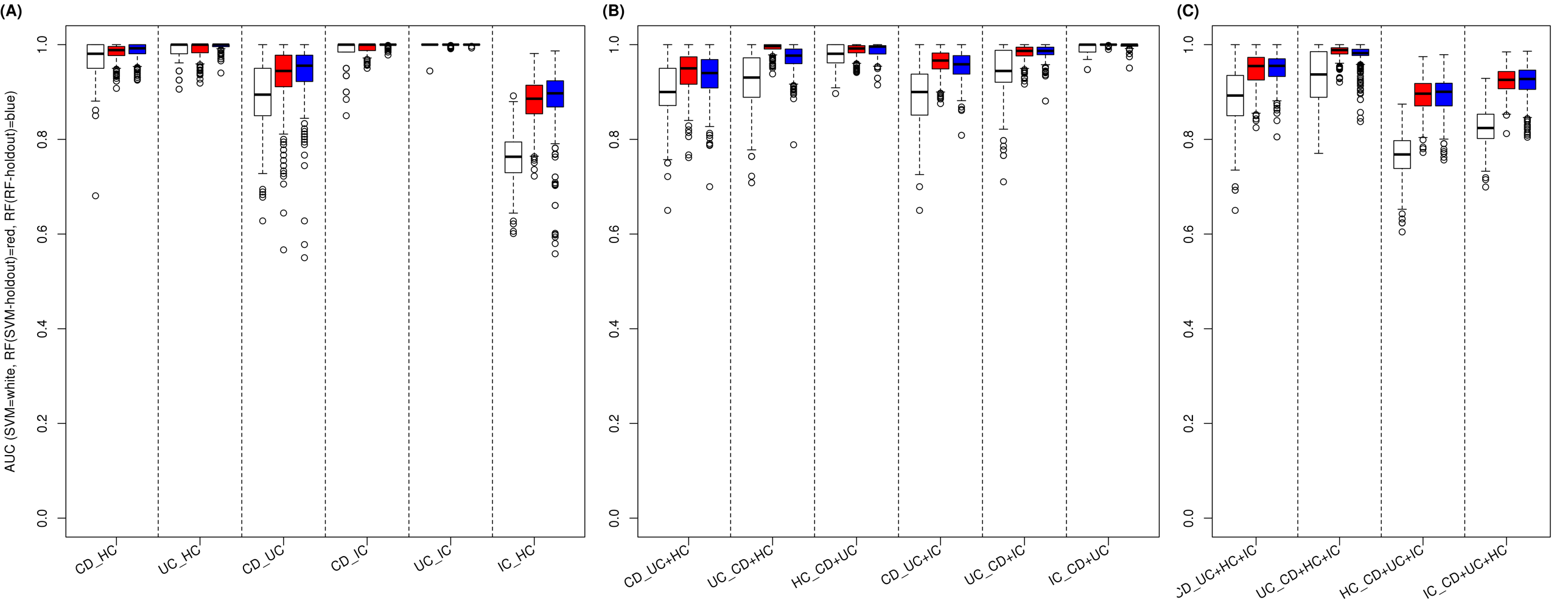

Supplement: S8 Fig — Measured by the area under the ROC curve (AUC) classification performance is shown for models considering (A) 2 groups (CD vs. HC, UC vs. HC, CD vs. UC, CD vs. IC, UC vs. IC, IC vs. HC), (B) 3 groups (CD vs. UC+HC, UC vs. CD+HC, HC vs. CD+UC, CD vs. UC+IC, UC vs. CD+IC, IC vs. CD+UC) and (C) 4 groups (CD vs. UC+HC+IC, UC vs. CD+HC+IC, HC vs. CD+UC+IC, IC vs. CD+UC+HC). Classification performance of the linear elastic SCAD SVM (white box) is compared to a Random forests considering variables selected using the median performing SVM (red box). Additionally, Random forests were trained with the top 50% of the variables ranked by their frequency of selection (blue box). (TIFF) [file pone.0140155.s008.tiff]
